# Building Accessible and Culturally Sensitive Resources: Alaska Native Community-Engaged Aging Research Lab

**DOI:** 10.1093/geroni/igaf122.1929

**Published:** 2025-12-31

**Authors:** Hannah Rebadulla, Murat Demir, Alexandra Timmins, Edmond Arroyo, Zoey Hilderbrand, Prince Alejo, Steffi Kim

**Affiliations:** University of Alaska Anchorage, Anchorage, Alaska, United States; University of Alaska Anchorage, Anchorage, Alaska, United States; University of Alaska Anchorage, Anchorage, Alaska, United States; University of Alaska Anchorage, Anchorage, Alaska, United States; University of Alaska Anchorage, Anchorage, Alaska, United States; University of Alaska Anchorage, Anchorage, Alaska, United States; University of Alaska Anchorage, Anchorage, Alaska, United States

## Abstract

Kim and colleagues joined with community members to develop accessible, community-driven online educational resources focused on dementia prevention, dementia knowledge, and dementia caregiver resources and support in remote Alaska Native communities. The presentation will outline community engagement strategies and efforts to strengthen community-held knowledge and practices that can be emphasized and built upon in Alaska Native dementia care practices and provide increased awareness of brain health and dementia knowledge. Presenters will outline the strengths and benefits of a collaborative that consists of various community partners engaging rural and remote communities related to aging, dementia, and caregiving. Furthermore, presenters will describe the unique engagement practices vital to sharing available resources and materials to rural villages in Alaska, including radio shows, attendance of cultural festivals, and community presentations.

